# Biophysics of magnetic orientation: strengthening the interface between theory and experimental design

**DOI:** 10.1098/rsif.2009.0491.focus

**Published:** 2010-01-13

**Authors:** Joseph L. Kirschvink, Michael Winklhofer, Michael M. Walker

**Affiliations:** 1 Division of Geological and Planetary Sciences, California Institute of Technology, Pasadena, CA 91125, USA; 2 Department of Earth and Environmental Sciences, Ludwig-Maximilians-University, 80333 Munich, Germany; 3 School of Biological Sciences, University of Auckland, Private Bag 92019, Auckland Mail Centre, Auckland 1142, New Zealand

**Keywords:** animal navigation, magnetoreception, geomagnetism, magnetite, radical pairs, double-blind protocols

## Abstract

The first demonstrations of magnetic effects on the behaviour of migratory birds and homing pigeons in laboratory and field experiments, respectively, provided evidence for the longstanding hypothesis that animals such as birds that migrate and home over long distances would benefit from possession of a magnetic sense. Subsequent identification of at least two plausible biophysical mechanisms for magnetoreception in animals, one based on biogenic magnetite and another on radical-pair biochemical reactions, led to major efforts over recent decades to test predictions of the two models, as well as efforts to understand the ultrastructure and function of the possible magnetoreceptor cells. Unfortunately, progress in understanding the magnetic sense has been challenged by: (i) the availability of a relatively small number of techniques for analysing behavioural responses to magnetic fields by animals; (ii) difficulty in achieving reproducible results using the techniques; and (iii) difficulty in development and implementation of new techniques that might bring greater experimental power. As a consequence, laboratory and field techniques used to study the magnetic sense today remain substantially unchanged, despite the huge developments in technology and instrumentation since the techniques were developed in the 1950s. New methods developed for behavioural study of the magnetic sense over the last 30 years include the use of laboratory conditioning techniques and tracking devices based on transmission of radio signals to and from satellites. Here we consider methodological developments in the study of the magnetic sense and present suggestions for increasing the reproducibility and ease of interpretation of experimental studies. We recommend that future experiments invest more effort in automating control of experiments and data capture, control of stimulation and full blinding of experiments in the rare cases where automation is impossible. We also propose new experiments to confirm whether or not animals can detect magnetic fields using the radical-pair effect together with an alternate hypothesis that may explain the dependence on light of responses by animals to magnetic field stimuli.

## 1. Introduction

During the past 50 years, suggestions that migratory and homing animals might use geomagnetic cues for navigation have moved from being a scientific fringe field to being a recognized discipline in animal behaviour and neuroscience. Initial scepticism was based both on the noisy and often irreproducible nature of the basic supporting experiments, the almost universal failure of conditioning experiments, as well as the perceived lack of a biophysical mechanism through which the weak geomagnetic field might lead to the controlled depolarization of a sensory nerve membrane. For example, the late Donald Griffin, co-discoverer of bat sonar, stated in his 1944 review of bird navigation that magnetoreception was biophysically impossible, owing to the lack of ‘physiological ferromagnetic materials’ (Griffin 1944). By 1969, however, he noted that the early work of Wiltschko and Lindauer provided compelling evidence for a magnetic effect on some behaviours, but was still puzzled by the lack of conditioning success, poor overall reproducibility and the mystery of the underlying biophysical mechanism (Griffin 1969). Evidence that an astounding variety of organisms do indeed respond to geomagnetic cues has accumulated since then, from small magnetotactic bacteria (Blakemore 1975; Kirschvink 1980) and protists (Bazylinski *et al*. 2000), all the way to migratory whales (Klinowska 1985; Kirschvink *et al*. 1986; Walker *et al*. 1992). Wiltschko & Wiltschko (1995) provide a thorough review of this work as of the mid-1990s.

Conditioning experiments were a tough nut to crack, but subsequent work demonstrated that most animals are more easily conditioned when they are presented with fields that are spatially distinctive (usually as a consequence of variations in magnetic intensity) and produce responses that require movement, which will expose the animals to the spatial variations within experimental spaces. This is the common feature for tuna swimming in big outdoor tanks (Walker 1984), free-flying honeybees (Walker & Bitterman 1985, 1989; Walker *et al*. 1989; Kirschvink & Kobayashi-Kirschvink 1991; Kirschvink *et al*. 1997), pigeons in flight cages (Mora *et al*. 2004), trout (Walker *et al*. 1997), zebra fish and tilapia (Shcherbakov *et al*. 2005) as well as sharks and rays (Kirschvink *et al*. 2001; Meÿer *et al*. 2005). It seems that the ability of most animals to respond to geomagnetic stimuli requires an environmentally relevant trigger to unlock or ‘release’ the behaviour (Walker *et al*. 2002). This is an important factor to remember in using such experiments to sort out proposed transduction mechanisms, as will be discussed below.

A fundamental tenet of neurobiology states that all information an animal obtains about environmental stimuli comes from cells specialized for transducing the stimuli and is conveyed by a coded stream of action potentials to the rest of the nervous system (see Block (1992) for an excellent review). This sensory processing is then used to influence the behaviour. Unfortunately, in studying magnetoreception, we most often are looking at some behavioural response that is a complex assemblage of many competing factors, from which we must infer the properties of the underlying biophysical reception and transduction mechanisms.

Over these years, a plethora of biophysical transduction hypotheses have been winnowed down to two or three that have experimental support: some form of electrical induction, specialized receptor cells involving biogenic magnetite (a strong ferrimagnet) and that of weak-field magnetic effects on photochemically generated radical pairs in the eye. Electrical induction has been largely eliminated as a mechanism in terrestrial animals on purely biophysical grounds, simply because the required anatomical structures would be large and easily visible; they simply do not exist (Rosenblum *et al*. 1985; Adair 1991). Recent work (see Kirschvink *et al*. 2001) has even shown that marine animals such as sharks and rays—which do have a highly developed electroreception system with macroscopic structures called the ampullae of Lorenzini (Kalmijn 1981)—do not appear to use it to detect the geomagnetic field as has long been suggested. This surprising discovery came from a simple experiment of fixing a permanent magnet to the snout of stingrays; a magnet moving with an animal should be invisible to an induction-based magnetic sensory system, yet the magnets abolished the magnetic discrimination whereas control brass weights did not (Kirschvink *et al*. 2001, but see Molteno & Kennedy 2009).

Fundamental to understanding the difficulties of studying the magnetic sense is the recognition that the magnetic fields to which organisms are typically exposed are very weak, which means the interaction energy between these magnetic fields and biological materials are typically orders of magnitude below the background thermal energy, *kT*. Because it was not clear how magnetic field effects on behaviour could be generated let alone demonstrated under the above circumstances, proposals for a mechanism of magnetic field detection based on interactions of electrons within biological molecules (and coincidentally for the effects of electromagnetic fields on health; Kirschvink 1997) were met with scepticism. The scepticism was compounded by the difficulty of achieving independent reproduction of positive experimental results (e.g. Reille 1968; Beaugrand 1976; Bookman 1977; Carman *et al*. 1987) obtained in both field and laboratory experiments. This combination of scepticism about the mechanism of magnetic field detection and the difficulties in reproducing results may have encouraged the use of methods that were known to work over pursuit of more powerful methods for studying the magnetic sense and its mechanism. In recent decades, magnetite, a magnetic mineral that interacts strongly with weak magnetic fields, has been identified in a range of animals (see Wiltschko & Wiltschko 1995; Kirschvink *et al*. 2001 for review), while more powerful methods for studying magnetic effects on behaviour in both the laboratory and field have become available. These more powerful methods now permit us to focus on achieving rigorous experimental designs that will permit rapid advances in analysis and interpretation of experimental results.

We suggest design of laboratory behavioural experiments should: (i) begin with the stimuli delivered to the subject animal in the experimental situation, (ii) ensure that the magnetic field stimuli provided are the only source of information that could be used by the animal to direct its responding in behavioural experiments, and (iii) make certain that the response produced by the animal is an unambiguous, measurable bit of behaviour that can be collected automatically wherever possible. Thus, care must be taken to achieve control of stimulation by ensuring that animals are exposed to only one of the two dimensions (intensity and direction) of experimental magnetic fields at a time and there are no extraneous cues (e.g. differential switching noise or magnetic intensity gradients present in an experiment investigating responses to magnetic field direction) that are correlated with changes in the stimulus being tested. Similarly, unambiguous behavioural responses that can be detected and counted automatically permit consistency of experimental control and data capture. Overcoming the combined challenges of achieving control of stimulation and robust control of behaviour by the experimental stimuli means that data obtained under such conditions are much less subject to biases. The data obtained in well-designed experiments are typically much easier to analyse and provide a stronger basis for interpretation of experimental results whether those results are positive, negative or inconclusive.

## 2. Experimental control and data acquisition in behavioural experiments

### 2.1. Generation of static and extremely low-frequency magnetic fields

The first consideration in laboratory studies of the magnetic sense is the Earth's magnetic field and its interaction with built structures in the experimental situation. Intensity, inclination and declination of the Earth's field vary so slowly in space that they are effectively constant on the scale of a laboratory, although they will be affected by time-varying fields due, for example, to the solar wind. The presence of structural iron in a building together with the fields produced by electrical equipment will interact with the Earth's field to cause variations in space (and time) in the observed field in a laboratory. Experimental spaces in which animals are studied should therefore be isolated as much as possible from spatial and temporal variations in the magnetic field owing to iron in buildings. This isolation can be achieved by locating the experimental space as close as possible to the centre (in three dimensions) of a room or, even better, in a purpose built structure that is free of magnetic materials such as iron (e.g. Fischer *et al*. 2001). For any behavioural experiment, the background field and experimental magnetic fields should then be mapped in detail with a fluxgate magnetometer, as the values within a building will often be different from those that exist outside the building.

**Figure 1.**
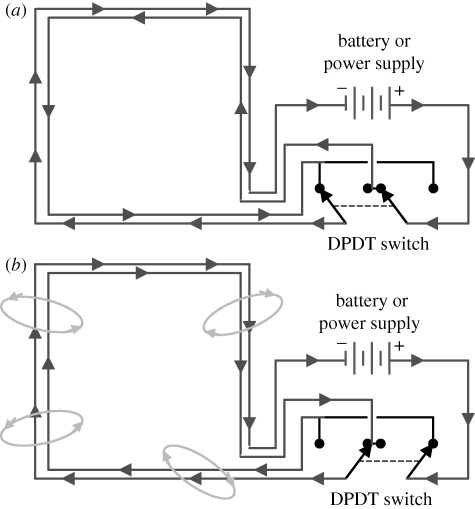
A simple circuit for introducing blind conditions in experiments using static or low-frequency magnetic fields, adapted from Kirschvink (1992*b*). When building the coil systems, wrap them carefully with two identical strands of wire, placed adjacent to each other and held firmly in place with high-conductivity thermal epoxy (to minimize vibration). These ‘double-wrapped’ coils can then be configured with a silent DPDT switch that will allow the current to flow in series through the two coils, but permits the direction of flow to be reversed in one of them. (*a*) Circuit with the DPDT switch set so that the current in the two coils is in opposition, yielding no external magnetic field (sham mode). (*b*) Same circuit with the switch set to produce parallel current flow, yielding the external magnetic field (active mode). We recommend using quasi-random Gellermann (1933) orders to set the active/sham states by a person external to the experiment. If that is impractical, a second DPDT switch can be inserted in the circuit, and two separate investigators can each control one of the switches with separate random orders to ensure fully blinded experimental conditions. Recommendations for multiple-coil designs to produce uniform magnetic stimuli within an experimental chamber are also given by Kirschvink (1992*b*).

Control of magnetic fields used in laboratory experiments can be required at several levels and can best be achieved using double-wrapped electromagnetic coils in which current flows in either parallel or antiparallel directions through the windings to generate an experimental field or no field, respectively (Beaugrand 1976; Kirschvink 1992*b*; shown schematically in figure 1). Such coils produce constant amounts of heat at constant current and so can have no influence on the outcomes of trials in which the experimental field produced by the coils is present or absent. Similarly, switching noise associated with the coils provides no information on the field present as any switching noise is consistent between experimental and control trials, can be kept isolated from the experimental space, and can use a silent (and hidden) double-pole, double-throw (DPDT) relay to switch the fields. Kirschvink (1992*b*) suggested extending this concept in combination with coil designs for producing large volumes of uniform magnetic fields that could be used in a variety of electromagnetic field (EMF) experiments in biology, as well as taking additional precautions like using high-conductivity thermal epoxy to eliminate subtle differences in the motions of adjacent wires between the active and sham states.

Control of induced fields in an experimental chamber requires coil systems that produce a uniform field space in experiments investigating compass orientation and conditioning to magnetic field direction. Although paired Helmholtz coils have often been used in such experiments, their uniform field spaces are very much smaller for a given coil size than those produced by designs using systems of three to five coils per axis (Kirschvink 1992*b*). The gradients present in magnetic fields produced by Helmholtz coils are relatively unlikely to affect outcomes of compass orientation experiments with migratory birds as the birds remain relatively close to the centre of the coil system. In directional conditioning experiments, however, a bird trained to choose a magnetic field direction by choosing a corner of an experimental arena will be exposed to the presence and absence of magnetic field gradients that can increase substantially as the bird moves away from the centre of the arena. As a consequence, there is potential for confounding of responses to field direction by response to field gradients as experimental subjects choose which corner of the arena they will enter.

An alternative strategy in experimental studies has been to condition animals to discriminate the presence and absence of localized magnetic intensity anomalies superimposed on the uniform background field. The anomalies introduce significant local variations in both magnetic field intensity and direction into the experimental spaces so it cannot be determined which of these dimensions of the field the animals are actually discriminating. The fields are produced by small magnetic coils associated with a response detector such as a micro-switch that the animal activates by pressing on a key or paddle or through some other behaviour that can be measured easily and objectively. An elegant coil system that uses two co-planar, concentric coils of equal dipole moment (area × current) but with antiparallel directions and different diameters (e.g. 2 and 10 cm; Kirschvink & Kobayashi-Kirschvink 1991) confines the anomaly to the area bounded by the larger of the two coils. This coil configuration has been used successfully to measure a threshold sensitivity to changes in the intensity of the anomaly at different frequencies (Walker & Bitterman 1989; Kirschvink *et al*. 1997) and could be used in combination with the large coils discussed above to carry out a range of psychophysical studies of the properties of the magnetic sense.

### 2.2. Generation of radio-frequency electromagnetic fields

**Figure 2.**
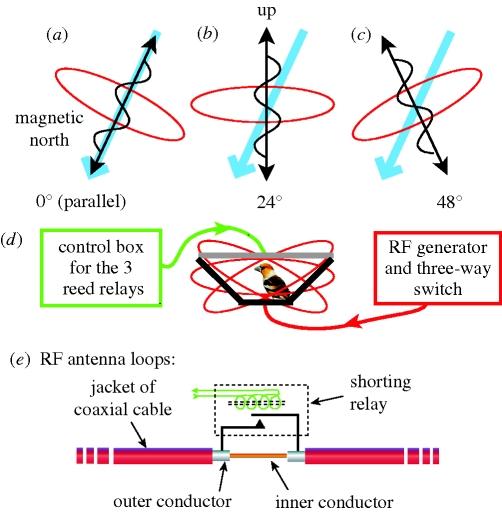
Suggestion for implementing a fully blind protocol on behavioural experiments involving both the static geomagnetic field and RF fields, adapted from the experimental protocol of Ritz *et al*. (2004). (*a*–*c*) The orientations of the antenna that were used by Ritz *et al*. (2004); the RF antenna is in red and the direction of the local geomagnetic field is shown in blue. The position of the RF antenna loop and the power setting on the RF generator (located immediately adjacent to the experimental chamber) are different in each condition (control or ‘sham’). Macroscopic differences other than RF parameters among various test conditions can be eliminated by replacing the mobile loop of coaxial cable with three identical loops fixed in the desired positions, and then using a remote switch to determine which coil is energized. (*d*) The alternative situation with three fixed loops centred on the bird's cage with a remote switch near the RF generator used to select one of the coils. (*e*) A method for silently shorting out the RF field using a small reed relay, activated by an external control box. The method is to short across the 2 cm stretch of the outer conductor (the ‘screen’) that is removed on the far side of the RF feed, thereby yielding no RF output for the control or sham. This allows the power output on the RF generator to be run at exactly the same levels for all experiments. By implementing these measures, experimental conditions would not be obvious to the investigators in the room, which otherwise could possibly affect the animals.

Ritz *et al*. (2004, 2009) report intriguing effects of radio-frequency (RF) magnetic fields of varying frequency which, in combination with the local geomagnetic field, can eliminate the magnetic compass response of migratory European robins, *Erithacus rubecula*. In their apparatus, a single loop of coaxial cable, 2.1 m in diameter, was modified to function as a circular antenna by stripping off 2 cm of the outer conductive shielding layer across from the RF feed. The coil was mounted on an assembly that allowed it to be rotated ±24° from the vertical, ranging from parallel to the local geomagnetic field, to vertical, to 48° from it, as illustrated schematically in figure 2*a*–*c* . The angle between the local geomagnetic field and that of the RF field was adjusted by physically rotating the antenna support structure to produce the desired angle between the geomagnetic field and the RF field before the birds were brought to the shed and placed in their funnels. The theory of radical-pair interactions predicts that the situation where the local geomagnetic field is parallel with the RF magnetic direction (perpendicular to the plane of the coil) should have no effect on behaviour, which is what was reported.

We note that the coil antenna was in three different positions in the different trials, and the RF power supply (which was located in close proximity to the experimental arenas) ran at different power levels in various trials. As birds are able to hear into both lower (Kreithen & Keeton 1974) and higher frequencies than humans and have broader visual abilities as well, subtle cues beyond the sensory abilities of humans might have been detected by the birds. Here we suggest a simple approach for eliminating macroscopic differences other than the RF parameters to exclude any potential confounding in RF exposure experiments. First, three separate antenna loops that are fixed in a permanent position (figure 2*d*) would obviate the need to rotate the antenna on a pivot for the three field settings. A three-way switch near the RF generator could then be used to direct the RF current to one of the coils and could easily be coded to obscure which one is active. Next, it is necessary to keep the RF generator itself operating at the same power level during all experiments. Our colleague, Prof. David Rutledge of the Caltech Radio Frequency and Microwave Group (see Rutledge 1999), suggested simply shorting across the 2 cm gap in the conductive shield of the coaxial cable used for the antenna, as shown in figure 2*e*. A short circuit across the gap eliminates the external field, but does not change the power drain on the RF amplifier at the low levels employed in these experiments. The short can be produced with a small relay located near the gap, controlled by an external switch. In fact, all three antennas can be wired to short with the same signal, as only one will be activated at any time. Even the acoustic noise of the tiny relays can be made symmetrical by using a series of paired relays, one shorting when activated, the other when passive, and then running them in opposition to produce the desired setting.

### 2.3. Control of experimental procedures

A focus on automation of behavioural experiments provides the opportunity for completely objective stimulation and recording of data as well as forcing a focus on the efficiency and effectiveness of experimental design. Fully automated procedures provide precisely timed control of delivery of a complex stimulus and reinforcement sequences while at the same time reducing variability in the recorded data by avoiding cueing or observer bias (Mora *et al*. 2009). Cueing (the ‘clever Hans’ phenomenon) occurs when an animal relies on extraneous cues arising from the experimental situation or cues unwittingly provided by the observer, rather than relying on the stimuli being tested in the experiment. Such automation also excludes the possibility of observer bias, subconscious reporting of favourable over unfavourable responses by the observer. Experiments using automated procedures successfully replicated many of the magnetic experiments on honeybees done by Walker and Bitterman, confirming the reality of the honeybee magnetic sensory system (Kirschvink & Kobayashi-Kirschvink 1991; Kirschvink *et al*. 1997). Even the sharp magnetic pulse—used to specifically test for the effect of remagnetizing single-domain biogenic magnetite—can be done in a blinded fashion using double-wrapped coils, as we did recently to demonstrate the ferromagnetic nature of the bat magnetic compass (Holland *et al*. 2008). Similarly, Mouritsen *et al*. (2004*a*) have significantly advanced the capture of data from birds hopping in funnel cages by video recording the bird's position. This approach permits automated measurement of bird movements within the orientation arena by image processing software and provides a permanent record of the behaviour.

To illustrate the benefits of careful control of stimulation in behavioural studies of the magnetic sense, we consider an experiment in which pigeons were successfully trained to discriminate the presence and absence of an induced magnetic anomaly in a conditioned choice procedure (Mora *et al*. 2004). To control for cues associated with switching artefacts, an array of resistors was used to substitute for the load on the power supply due to the coils. Although the birds showed no evidence for detection of switching noise, a possibility of doubt remained because cues associated with the coils could have been used by the birds to predict the delivery of food and to respond accordingly. Had the coils been double wrapped as suggested above, the possibility that differences due to the presence and absence of current passing through the coils would have been explicitly excluded as an explanation for the behaviour.

In contrast, a study that attempted to replicate the reported effects of static magnetic fields on cryptochrome (CC)-dependent responses in the plant *Arabidopsis thaliana* (Ahmad *et al*. 2007) and used double-wrapped coils implemented fully blind protocols for all molecular analyses but was unable to replicate the claimed magnetic effects (Harris *et al*. 2009). The above arguments and evidence all affirm the need to use the best available techniques to ensure reliable results in magnetoreception experiments.

## 3. Magnetite versus radical-pair-based magnetoreception: a logical comparison

In this section, we ask whether or not the published data on optical effects on magnetic compass orientation can be explained only by the presence of two different biophysical mechanisms for magnetic field transduction in animals as has been implied (magnetite and a radical-pair compass (Phillips 1986; Wiltschko, R. *et al*. 2007)) or whether the data are compatible with a set of magnetite-based receptor cells, the responses of which are combined with other sensory inputs to yield the observed behaviour.

**Figure 3.**
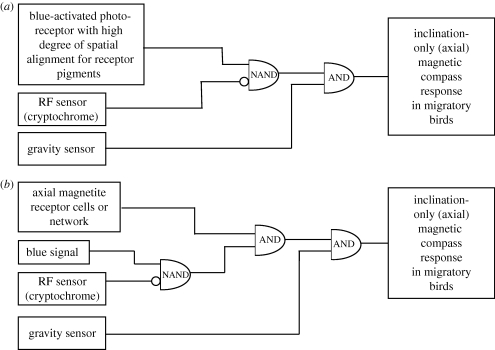
Logical information flow diagrams for the axially symmetric, blue-light-activated, RF-inhibited migratory magnetic compass response observed in European robins and studied by Ritz *et al*. (2004). Boxes on the left indicate the primary receptor cells that transduce the indicated stimulus into a coded stream of action potentials. These are usually processed locally, then sent via the major sensory nerve bundles and intermediary ganglia to the brain, represented here by simplified logic gates. The AND gate is a logical construction that passes information (left to right) only if both of the two inputs are true, and the bubble is the standard symbol for an inverted signal. (True becomes false and vice versa; true/false in this context means receiving/lacking streams of action potentials from the receptor cells, indicating the presence/absence of the appropriate activating cue.) (*a*) Logical flow diagram for the hypothesized radical-pair magnetic compass. (*b*) Similar logical flow diagram for an axial, magnetite-based compass.

Figure 3 shows these two interpretations diagrammatically, in terms of logical flow circuits. Boxes on the left side of the figure represent the primary sensory modalities—the specialized receptor cells and perhaps a few adjacent neurons that process the information locally. The right sides of the diagrams indicate the observed behavioural response, which for this discussion is specifically the axially symmetric, blue-light activated, RF-inhibited migratory magnetic inclination compass response observed in European robins as reported by Ritz *et al*. (2004). (The polar, fixed-direction magnetic compass is discussed separately below.) The half-oval symbols are AND gates, the output of which are activated (true) if and only if both of the inputs are also true. (True/false in this context means receiving/lacking streams of action potentials from the receptor cells, indicating the presence/absence of the appropriate activating cue.) In both models, we have separated the RF sensitivity from the blue-light sensitivity, as they are quite different stimuli even though they may be transduced by the same radical-pair molecule. However, unlike the blue-light detector the RF sensor acts in an inhibitory mode, so its connection to the first gate is shown with an inverting bubble, converting the logical gate to a type of ‘NAND’ junction. Both excitatory and inhibitory synaptic connections are well known in neurobiology.

Figure 3*a* is a logical map of the CC variant of the radical-pair compass. In order to achieve a compass, it is necessary that the blue-light-sensitive pigment molecules (possibly CCs) are aligned physically within each receptor cell so that their net interaction with the geomagnetic field at the cellular level can be detected, which is a condition for an axial magnetic compass (Schulten 1982; Ritz *et al*. 2000). In figure 3*a*, the output of this system goes directly into the first NAND gate, as the presence of blue light enables the compass behaviour. Similarly, as the presence of certain frequencies and directions of RF radiation inhibit the response, the presence of RF radiation is shown as inhibiting the behaviour (via the inverting bubble on the connection to the NAND gate). Finally, a gravitational component is essential for the operation of the bird's inclination compass, so the presence of a neurological input from the vestibular system is necessary to release or activate the observed behavioural response.

Figure 3*b* shows an alternative interpretation that explains exactly the same data. In this model, the magnetic field axis is determined by a small array of magnetite-containing cells configured to yield the field direction (not the polarity—see §4). The output of this system is again enabled by the presence of blue light and inhibited by the presence of the RF signal, as above. However, in this model, there is no need for the blue signal to come from an *ordered* array of pigment molecules, although the same molecule (such as a CC) could be responsible for both the blue response and the RF sensitivity. Only one or at most a few cells sensitive to blue light and RF magnetic fields would be needed, as the response is a simple signal indicating the presence or absence of blue light. The actual magnetoreceptors could be located anywhere, even in the retina (which does contain ferromagnetic material in some animals, as noted in several chapters in Kirschvink *et al*. (1985*a*)). A possible evolutionary scenario for the optical effect on a magnetite-based magnetic compass is described in the electronic supplementary material.

As of the time of writing, we know of no experimental data that can unequivocally distinguish between the two interpretations of the behaviour, a conclusion also supported by a recent analysis by Jensen (2010). However, there are obviously more logical ‘gates’ in the animal's behavioural programming, as we have not included such things as the narrow magnetic intensity windows (which would presumably need inputs from a magnetic intensity detector), or the generally lower activity of some animals in total darkness.

## 4. Discussion

Not only is the study of the magnetic sense and its use by animals inherently multi-disciplinary, crossing physics, bio- and geo-physics together with associated structure and function of sensory receptor cells, nerves and associated brain areas, it must also be carried out across scales from atomic (radical pairs) to inter-hemispheric distances and nanoseconds to years in time. It can be difficult to understand and apply the theory and approaches of multiple disciplines effectively and to analyse and interpret well the results of experiments that cross disciplinary boundaries. When such work is to be published, it is reasonable to expect that the contributions from different disciplines will be both persuasive to experts inside those disciplines and intelligible to readers from outside them. The separate components of work must then be integrated well in order to be persuasive to researchers interested in the specific problem.

We suggest that the challenge of meeting the above requirements is increased by both the lack of a magnetic sense in humans and the difficulty of using behavioural approaches to identify a sensory mechanism, as has occurred in the case of the magnetic sense. Because magnetic fields pass through tissue, no accessory structure such as a lens or ear canal is required to bring the external magnetic field inside the body and project it onto a sensory epithelium. That is, there does not need to be a specific ‘magnetic sense organ’—magnetoreceptor cells to be distributed throughout the body. An accessory sensory structure, however, invites structural study and facilitates the identification of a mechanism, whereas behavioural methods permit highly detailed study of the capacities of sensory systems. In the discussion that follows, we examine the results obtained from behavioural and some structural studies.

### 4.1. Magnetite

Magnetite biomineralization is a well-known phenomenon, having been first discovered in the linings of the major lateral teeth of the Polyplacophoran molluscs by Lowenstam (1962), who suggested that it might help guide their homing instinct because it is ferrimagnetic. Subsequent biophysical analyses have shown that biogenic magnetite is a viable mechanism for magnetoreception (Kirschvink 1979; Yorke 1979; Kirschvink & Gould 1981; Kirschvink & Walker 1985; Kirschvink 1992*a*). Magnetite crystals perfectly suited for magnetoreception have been found in all domains of living organisms except the Archaea, with a variety of candidate receptors in animal tissues (see Kirschvink *et al*. (2001) and Walker *et al*. (2002) for more recent work). Numerous experiments, developed for animals by Kirschvink *et al.* (1985*b*) following the elegant experiment on magnetotactic bacteria by Kalmijn & Blakemore (1978), have shown conclusively that at least some magnetoreceptors are indeed based on ferromagnetism (Kirschvink & Kobayashi-Kirschvink 1991; Beason *et al*. 1997; Wiltschko, W. *et al*. 2002, 2007, 2009; Holland *et al*. 2008), either the magnetite/maghemite solid solution or (less likely in most animals) greigite. In fact, one of the simplest magnetite-based magnetoreceptor models is consistent with the known ultrastructure in fish (Walker *et al*. 1997; Diebel *et al*. 2000): a magnetosome chain anchored to the nerve membrane near mechanically activated ion channels.

On theoretical grounds (Kirschvink & Gould 1981; Winklhofer & Kirschvink 2010), a magnetite-based compass magnetoreceptor system permits a species to have a compass that operates in either a polar or axial (inclination) mode as well as a fixed-direction response (see below). In view of this, the observation of inclination-only magnetic compass responses in many animals does not help to distinguish between a magnetite-based system and one using radical pairs. On the other hand, the polar compass in mole rats (Marhold *et al*. 1997; Thalau *et al*. 2006), bats (Holland *et al*. 2008) or arthropods (Kirschvink & Kobayashi-Kirschvink 1991; Lohmann *et al*. 1995) is consistent with a magnetoreception mechanism based on magnetic remanence (e.g. single-domain magnetite).

A ‘fixed-direction’ response (Wiltschko, R. *et al*. 2007) is observed when birds placed in an orientation arena are exposed to a combination of 502 nm turquoise and 590 nm yellow light, without the intermediate wavelengths that elicit the migratory response discussed in figure 3. The fixed-direction response is polar in nature (the birds can distinguish inversion in the vector field direction, from **B** to –**B**), does not depend on light to occur (Stapput *et al*. 2008) and moves with shifts in the local magnetic field direction (making it a compass response requiring a specialized receptor cell). The response is abolished by anaesthesia of the area of the upper beak in birds (Wiltschko, R. *et al*. 2007) where superparamagnetic magnetite is located inside dendrites (Winklhofer *et al*. 2001; Fleissner *et al*. 2003). The iron-bearing dendrites on the other hand are not involved in the light-sensitive inclination compass (Zapka *et al*. 2009). Curiously, the fixed-direction response has higher variance in the presence of RF magnetic fields (see fig. 1 of Wiltschko, R. *et al*. (2007) and fig. 3 of Stapput *et al*. (2008)). This observation suggests some RF sensitivity that does not depend on the presence of light, while all the other results above indicate that the fixed-direction response is most likely to reflect a magnetoreception mechanism based on magnetic remanence, which would provide a polarity bias, but not on superparamagnetic magnetite, which by definition has no remanence and behaves axially. Tian *et al*. (2007) magnetically detected remanence-bearing material besides superparamagnetic particles in the upper beak skin of homing pigeons (fig. 1 of Tian *et al*. (2007)). The distinct magnetic behaviour at 120 K, which corresponds to the Verwey transition temperature of pure magnetite shown in fig. 3 of Tian *et al*. (2007), implies that the magnetic remanence in beak samples is due to magnetite. So far, however, magnetite crystals large enough to carry magnetic remanence have not been detected in the iron-bearing dendrites of the upper beak skin (Hanzlik *et al*. 2000; Winklhofer *et al*. 2001; Fleissner *et al*. 2003), while they have been extracted from the ethmoid (Walcott *et al*. 1979).

**Figure 4.**
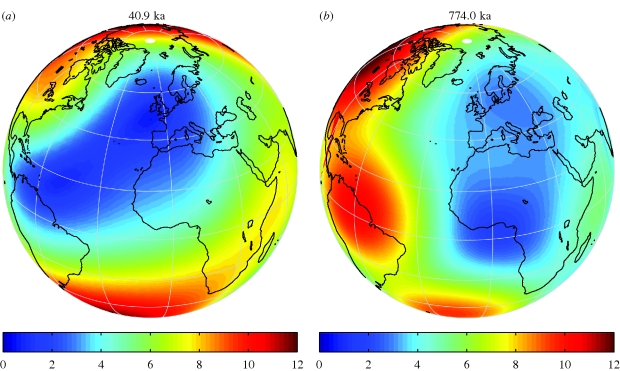
Geomagnetic field intensity (in µT) during European climax of (*a*) the Laschamp magnetic excursion and (*b*) the last geomagnetic reversal (Brunhes/Matsuyama). The snapshots for the indicated times were computed from the spherical harmonic coefficients provided in Leonhardt *et al*. (2009) and Leonhardt & Fabian (2007), respectively. The indicated times are in thousands of years (Kyr) before present. (*a*) At the climax of the excursion, the field in Europe was generally less than < 4 µT with values as low as 2 µT along the southwestern bird migration corridor to Morocco (Northwest Africa). (*b*) This situation is similar to that of the last geomagnetic reversal, at the climax of which Europe experienced fields generally weaker than 4 µT. These periods of very low field strength last of the order of a few centuries (see also electronic supplementary material).

On evolutionary grounds, it would not be surprising to find more than one flavour of magnetite-based receptor cell in animal tissues. Evolution is very good at taking an existing system (such as an ancestral sensory cell), replicating it via gene-duplication events and allowing the duplicated system to evolve new functions; this is called evolutionary ‘exaptation’ (Gould & Vrba 1982). This has clearly happened in many sensory systems, including the evolution of colour vision, frequency sensitivity in hearing, a plethora of flavour-specific taste buds, etc., so it would not be surprising to find the same pattern expressed in magnetoreception. One could easily see how natural selection could result in differing cellular magnetic moments for different functions such as intensity reception, the ability to function during geological intervals with weak magnetic fields (figure 4), as well as structural changes that give polar versus axial magnetic compass signals. Magnetite biomineralization is an ancestral trait to the entire animal kingdom, present even in the protist ancestors of the first multi-cellular animals (Bazylinski *et al*. 2000). Even some of the proteins that are involved in magnetite biomineralization in the magnetotactic bacteria still retain the capability to induce magnetite formation in animal tissues, such as Mag-A when introduced to mammalian cells (Zurkiya *et al*. 2008). The magnetite biomineralization system may well be ancestral to other matrix-mediated biomineralization pathways, as suggested by the ‘Grand Unified Theory of Biomineralization’ (Kirschvink & Hagadorn 2000). Hence, we have no *a priori* reason to suspect that these receptors would be localized in only one area or tissue type.

### 4.2. A radical-pair compass

Effects of moderate-strength magnetic fields on radical pairs are a real phenomenon (see Rodgers & Hore (2009) for a recent review). The current candidate protein to host magnetically sensitive radical-pair reactions is CC (Ritz *et al*. 2000), which has been identified in displaced ganglion cells in the retina of migratory birds (Mouritsen *et al*. (2004*b*); see also the review by Liedvogel & Mouritsen (2010)). The radical-pair compass hypothesis can be split into two separate related hypotheses, as follows.

1. The geomagnetic field modulates the natural, hyperfine-field-driven singlet to triplet interconversion (and vice versa) in a radical pair under physiological conditions and the corresponding chemical signal (magnetically induced change in singlet/triplet yield) is transduced to the nervous system in a specialized sensory cell.
2. A spatially and temporally coherent alignment of radical-pair host molecules within this sensory cell can exploit the axial anisotropy of the radical-pair reaction to extract information about the axial orientation of the external magnetic field.

The ‘smoking gun’ evidence for the radical-pair magnetic effect directly supports hypothesis 1. In Ritz *et al*. (2004, 2009) and Thalau *et al*. (2005, 2006), it was demonstrated exquisitely that even a weak RF magnetic field (in the 1–10 MHz range) has an effect on the orientation behaviour of migratory birds (but not on mole rats, a poikilothermal rodent adapted to life in the subsurface), provided that the magnetic vector of the RF field was applied at an oblique angle with respect to the static field. When both magnetic fields were parallel, there was no (or minimal) RF effect. So far, we know of no magnetic biominerals or biological molecules other than a radical pair that would display a similar anisotropic behaviour in the low megahertz frequency range. Since the hyperfine coupling strength in a radical pair corresponds to a frequency in the low megahertz range, the application of a megahertz field should interfere with the natural hyperfine-driven singlet–triplet interconversion (Ritz *et al*. 2004). Although *in vitro* studies on synthetic radical-pair molecules condensed into a liquid-crystalline phase have demonstrated the feasibility of a chemical compass sensor (Maeda *et al*. 2008), there is no ultrastructural support for hypothesis 2. Also, as we have discussed above (logical network diagrams), a positive test of hypothesis 1 does not necessarily imply that hypothesis 2 is true. While a coherent alignment of proteins that host the radical-pair reaction is needed for the compass to work, it is not needed to explain the observed effects of the RF field. This was also shown in Rodgers *et al*. (2005), where the electron-paramagnetic resonance spectra of a synthetic radical pair depends strongly on the relative orientation of RF field and static field, although the molecules were in a solution at room temperature and thus subject to Brownian rotational motion.

Lending experimental support to hypothesis 2 is actually a difficult problem. In the following, we focus on some important ultrastructural and biophysical features that must exist if directional information about the magnetic field is to be extracted from the basic radical-pair interaction. Although the spins in the radical pair are not strongly coupled to the thermal bath (Ritz *et al*. 2004), the proteins that host radical-pair reactions are subject to Brownian motion and therefore must be tethered to larger subcellular components that provide a fixed frame of reference for the compass. Schulten (1982) and Ritz *et al*. (2000) assumed the molecules that host the radical-pair reaction to be anchored to a biological membrane. However, biological membranes do not form stable reference platforms on the submicrometre scale. Detailed biophysical calculations of thermal fluctuations have shown that, for a typical eukaryotic membrane held in place with a cytoskeletal system, the membrane surface constantly wiggles around, with a broad distribution of surface tilts up to 90° (Brown 2003). It is therefore not as stable as commonly assumed. The only way to dampen such membrane warps is by stiffening the membrane or by increasing the viscosity of the cytosol (e.g. Tuvia *et al*. 1997). However, the margin to increase the viscosity is quite limited, given that physiological fluid viscosities (including that of cytosol) range between 1 and 4 cP (e.g. Tuvia *et al*. 1997). Higher viscosities would have other adverse effects on the cell, such as slowing down nutrient flow, oxygen diffusion, intracellular traffic and transduction pathways.

Mouritsen *et al*. (2004*b*) suggested that, owing to its cytosolic nature, CC in retinal ganglion cells could be fixed to the cytoskeleton. A tether connecting CC and cytoskeleton would have to be short, where short means much smaller than the thermal persistence length of the tether, *L*_t_ *= EI/kT*, where *E* is the Young modulus and *I* is the second moment of inertia of the tether. For example, for actin filaments, *L*_t_ is 15 µm (e.g. Howard 2001, p. 111). A tether with length *L* > *L*_t_ is continuously bent by thermal forces (two rotational degrees of freedom), whereas one with length *L* << *L*_t_ behaves like a stiff rod. Thus, by choosing *L* << *L*_t_, two degrees of rotational motion (bending or wagging) are effectively attenuated, and the only remaining degree of freedom can be activated by twisting the filament. The twisting angle *ϕ* induced by a thermal torque of magnitude *kT* is given by
4.1
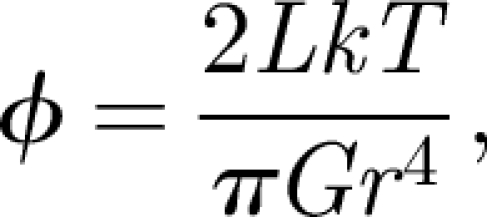

which for actin (*r* ∼ 3 nm, *G* ∼ 1 GPa) is 2*L* × 10^−3^ deg nm^−1^. Again, as long as *L* << *L*_t_, twisting is negligible. The filament to which the protein is connected on one end may be anchored to the cell membrane on its other end, in which case the filament–protein complex is subject to shape fluctuations of the membrane. It is therefore better to anchor the molecules to an interconnected filament network.

In principle, Brownian rotational motion can be largely blocked out by condensing or crystallizing CC proteins tightly into a vesicle. Although this slows down possible transduction pathways, it may be a good way to sequester superoxide—the assumed partner of the pigment cofactor in CC (Ritz *et al*. 2009)—from the cytosol where it is prone to cause damage. Transmission electron microscopy and small-angle diffraction studies have the potential to reveal ordered arrays of receptor molecules if present.

Last, we consider the case of unconstrained CC proteins free to rotate. Since the result of free rotational diffusion is thermal randomization of the orientation of the molecular axis relative to the magnetic field axis, this case is not directly relevant to the hypothetical radical-pair compass. However, it provides a useful estimate of the minimum time constant *τ* of rotational diffusion (or, conversely, the maximum diffusion coefficient *D*). This in turn can be used for a rapid experimental screening technique on the basis of measurements of *τ* in live preparations of retinal ganglion cells such as those identified by Mouritsen *et al*. (2004*b*). This should help to distinguish those types of cells containing untethered, rapidly diffusing CC molecules from proper candidate cells containing suitably anchored CC with much longer diffusion times so as to narrow down the search for target cells for electrophysiological recordings. Between time *t*_0_ and *t*, the mean square angular deviation 
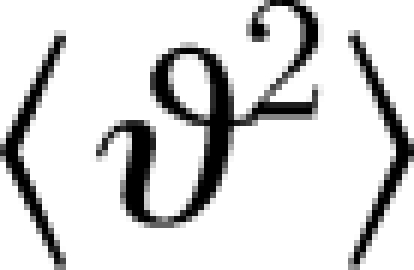
 of a molecule due to Brownian motion increases by
4.2
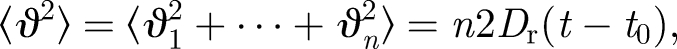

where *D*_r_ is the rotational diffusion coefficient of the molecule and *n* is the number of rotational degrees of freedom, e.g. about how many axes the molecule can diffuse. Cytosolic CC, if not tethered, has *n* = 3 degrees of rotational freedom. The first rotational diffusion coefficient *D*_r_ has not been experimentally determined yet (to our knowledge). A first rough guess of *D*_r_ for CC can be obtained from scaling the *D*_r_ value determined on lysozyme (∼15 kD molecular weight, *D*_r_ ∼ 20 rad^2^ µs^−1^ at 20°C (Cross & Fleming 1986)), assuming similar density and comparable aspect ratios. This scaling yields a first estimate of *D*_r_ ∼ 5 rad^2^ µs^−1^ for CC (molecular weight ∼60 kD; Liedvogel *et al*. 2007). Since the dimensions of the CC molecule are similar to those of photolyase (80 × 60 × 30 Å^3^; Sancar 2000), we can also forward calculate *D*_r_ by assuming that its shape can be approximated by that of a general ellipsoid. From expression (36) in Jeffery (1922), we calculated the three diagonal rotational coefficients *D*_*a*_, *D*_*b*_ and *D*_*c*_ of the CC molecule as 6, 4.5 and 4.1 rad^2^ µs^−1^, respectively, at body temperature (38°C) and a viscosity of *η* = 1 × 10^−3^ kg m^−1^ s^−1^ (=1 cP in cgs), assuming the CC molecule, a water-soluble protein, to have a solvation shell of 4 Å. Thus, the mean rotational diffusion coefficient, *D*_r_ = (*D*_*a*_ + *D*_*b*_ + *D*_*c*_)/3, amounts to 4.9 rad^2^ µs^−1^, which agrees very well with the rough estimate of *D*_r_ on the basis of the molecular weight. A *D*_r_ value of 5 rad^2^ µs^−1^ corresponds to a typical diffusion rate (or relaxation time) *τ* = 1/(6*D*_r_) ∼ 0.03 µs, which may be determined with fluorescence depolarization measurements on unfixed tissue preparations, by labelling CC with a high-quantum yield, high-emission anisotropy fluorophore with sufficiently long-lived fluorescence lifetime *τ*_F_ (where *τ*_F_ should at least be five times greater than the expected rotational correlation time *τ* of CC). Diffusive rotation of CC molecules in the time interval between excitation with a linearly polarized light pulse at time *t*_0_ and emission at time *t* depolarizes the emitted light relative to the excitation light and thus diminishes emission in the original polarization direction while it reinforces emission in the two orthogonal directions. Additionally, flash recovery after photobleaching on live preparations can be used to determine directly the ability of CC to diffuse *in vivo*. In this procedure, which can be done on many confocal microscope systems, all CC molecules within a set area within one of the ganglion cells would be ‘bleached’ by a strong flash of blue light, tailored to the absorption peak of CC. The rate at which unbleached molecules diffuse into the bleached zone is a measure of their translational diffusion ability. Translational diffusion in this context would also imply rotational diffusion. CC constrained enough to function as a compass should diffuse neither in translational nor in rotational modes. This would provide immensely important information about the suitability of the candidate molecules in a magnetoreceptor cell that is supposed to provide compass information.

To conclude, a radical-pair compass requires both spatial and temporal coherence in the orientations of the CC molecules in the displaced ganglion cells, which in turn requires structural links, preferentially to a filament network. The structures that hold CC in a coherent array in the displaced ganglion cells may be seen by staining compared with control cells, such as other non-CC ganglion cells, rods and cones. Similarly, as the cells are known, they can presumably be located in an isolated retina, so it should be possible to record from them. While perfect structural order is not strictly required (Winklhofer 2009; Hill & Ritz 2010; Lau *et al*. 2010), the effect of the thermal bath remains a challenge, even in the network case. Model calculations by Lau *et al*. (2010) suggest that, for a radical-pair compass to be viable, rotational correlation times should be an order of magnitude longer than for freely rotating CC proteins in cytoplasm.

## 5. Conclusions

Substantial progress in understanding the structure and function of the magnetic sense has been achieved in recent decades but there is still a very long way to go before we can claim to understand the sense in any detail. At this point, there are intense debates over proposed mechanisms of magnetic field detection that almost certainly reflect the paucity of knowledge on structure and function at the level of the detector cells themselves. We suggest the ambiguities in the interpretation of experimental results (as discussed in §3) and difficulty in determining the mechanism(s) of detection of magnetic fields reflect the limited power of behavioural methods to address questions of the identity of sensory mechanisms. We suggest that future studies of the mechanism of the magnetic sense should focus on the structure and function of the cells that are hypothesized to detect magnetic fields.
